# Supplementary figures and images for: A new genus of oryzomyine rodents (Cricetidae, Sigmodontinae) with three new species from montane cloud forests, western Andean cordillera of Colombia and Ecuador
Source: PeerJ. 2020 Nov 10;8:e10247. doi: 10.7717/peerj.10247 (PMC7664470; doi:10.7717/peerj.10247)

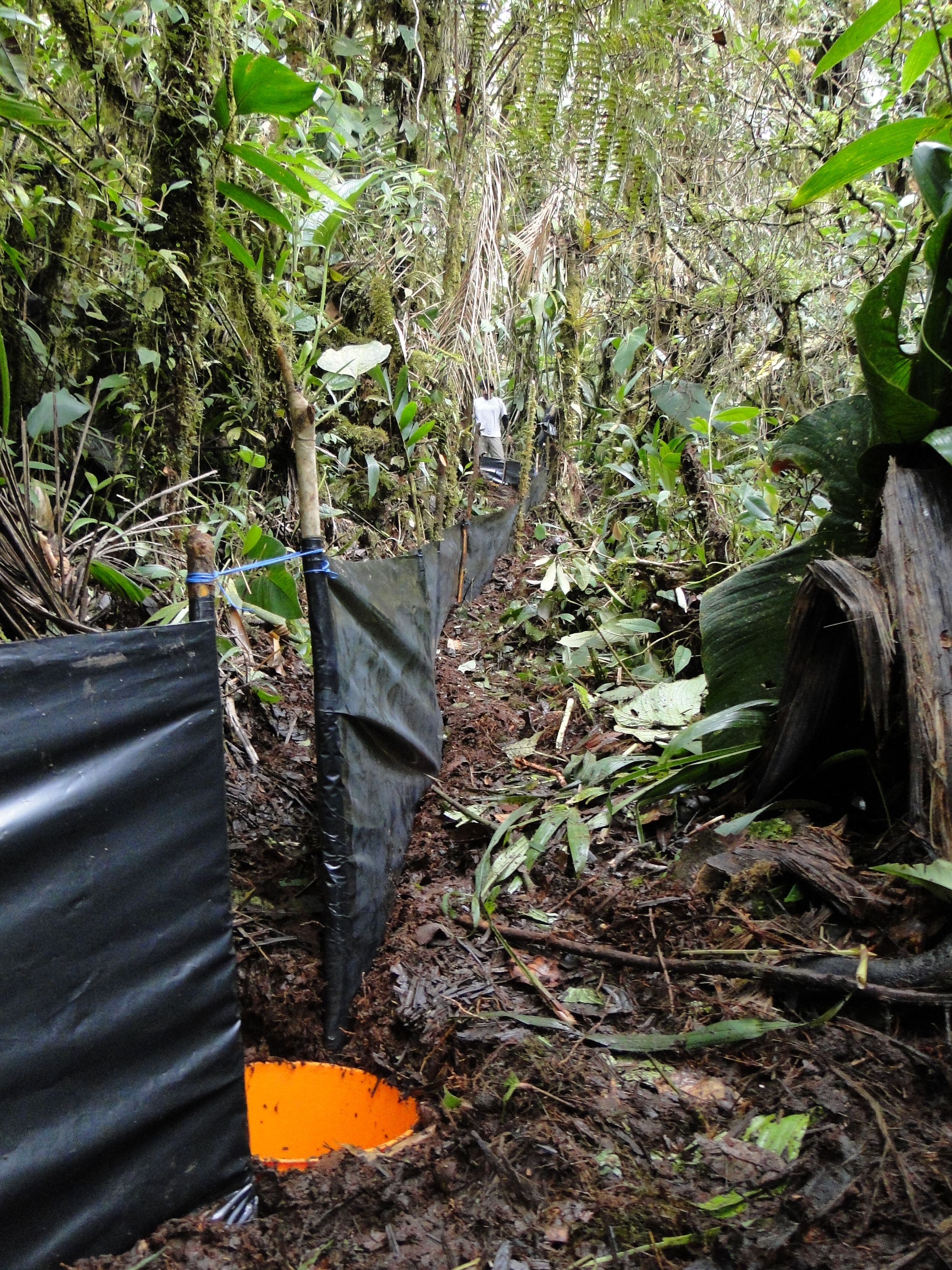

Supplement: Supplemental Information 1 — Pitfall line, the main technique for capturing small mammals in this study (Reserva Drácula; photo: J. Brito). [file peerj-08-10247-s001.jpg]

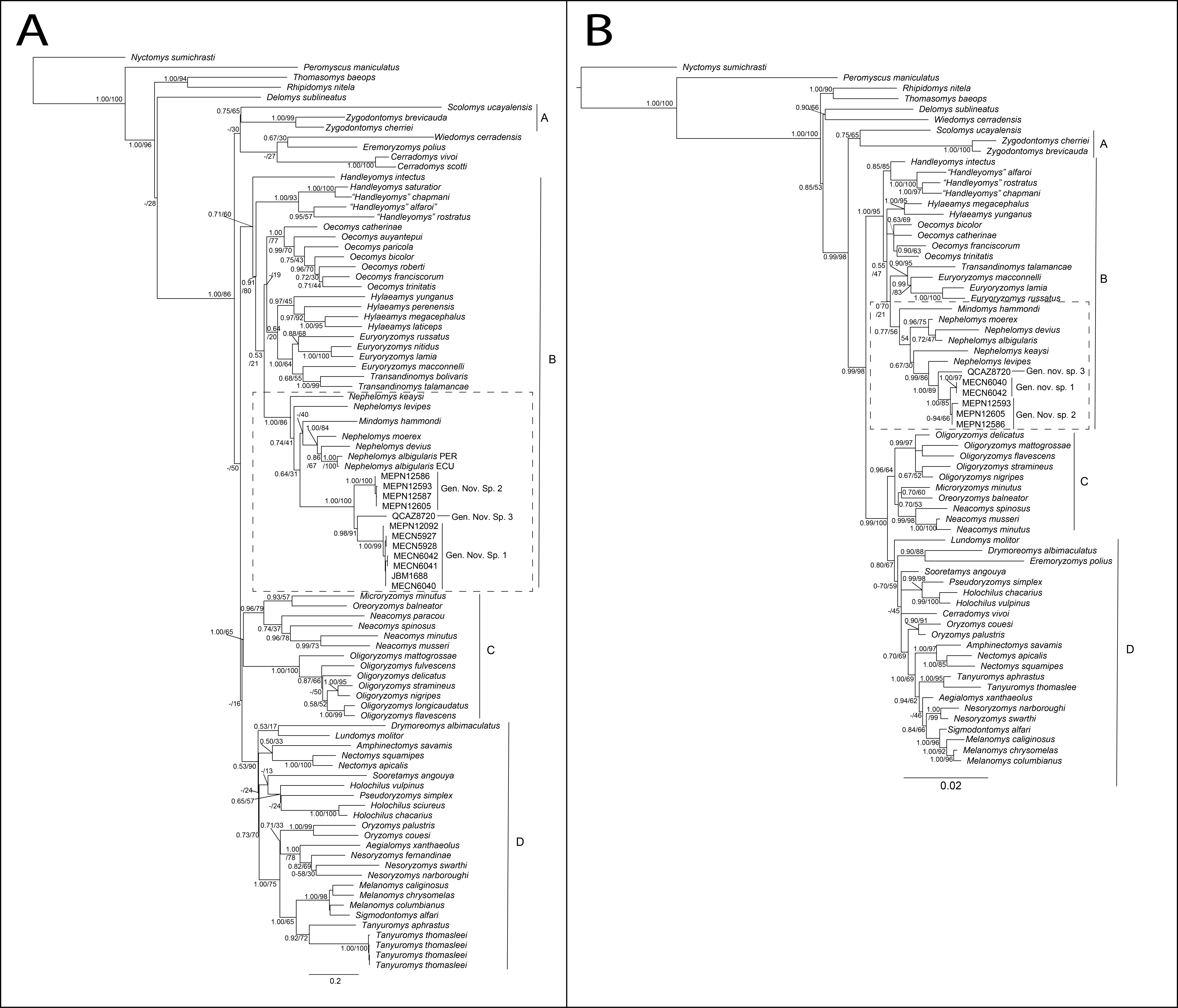

Supplement: Supplemental Information 5 — Best tree from ML analyses from analyses of DNA sequences of mitochondrial Cytb (A) and nuclear IRBP (B) genes. Numbers below branches are ML bootstrap support and posterior probability values. Clades are indicated by letters (A to D). The dash line indicated the position of new taxa. [file peerj-08-10247-s005.png]

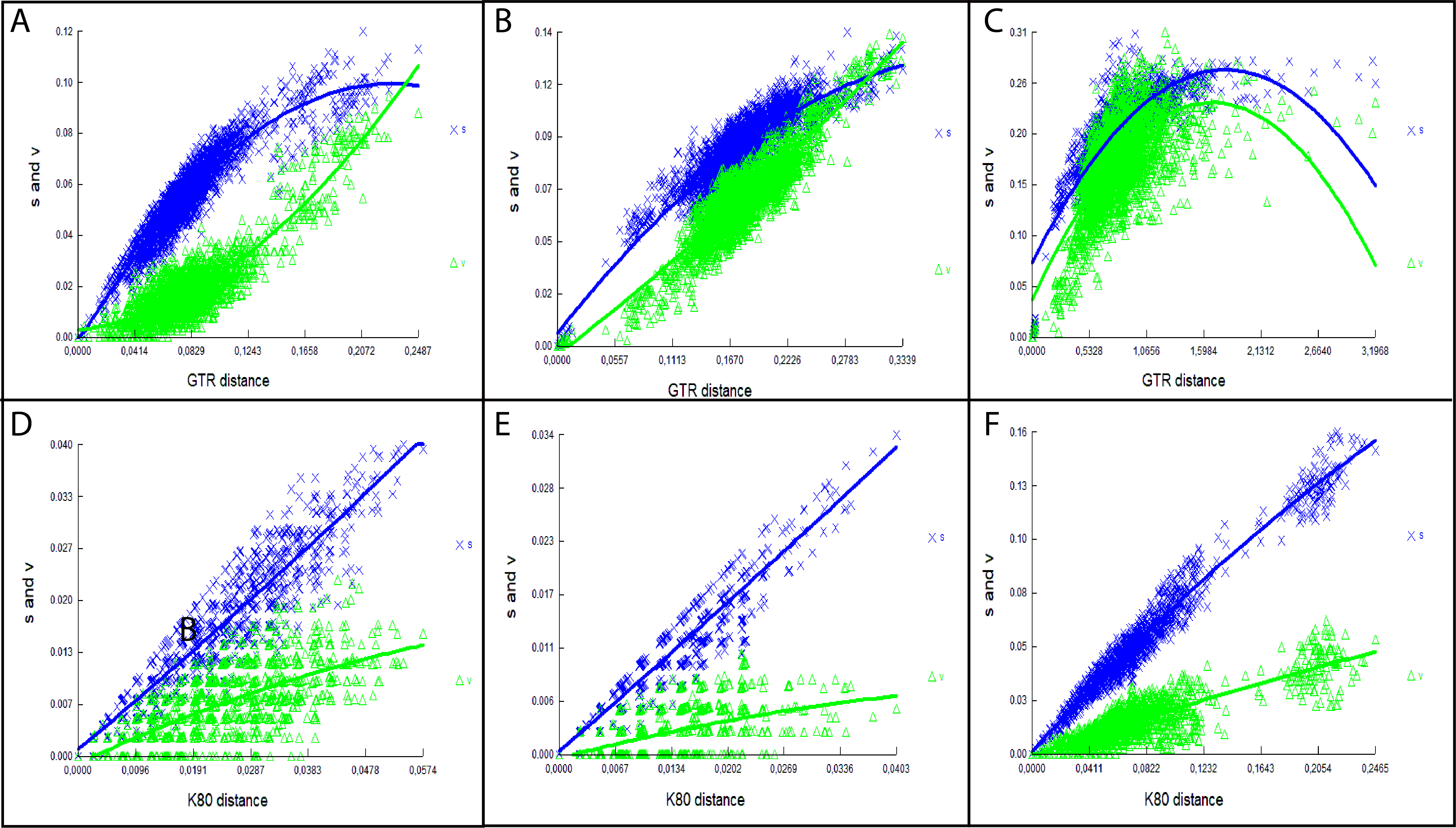

Supplement: Supplemental Information 6 — The x-axes GTR for Cytb and K80 for IRBP distance is based on the GTR for Cytb and K80 for IRBP substitution model and is expected to increase linearly with divergence time. The vertical axes is for the observed proportion of transitions (s) and transversions (v), respectively. Graphs A, B and C represent the results of saturation of the first, second and third positions of the mitochondrial gene Cytb (Cytb) and graphs D, E and F represent the results of saturation of the first, second and third positions of the nuclear gene IRBP. [file peerj-08-10247-s006.png]

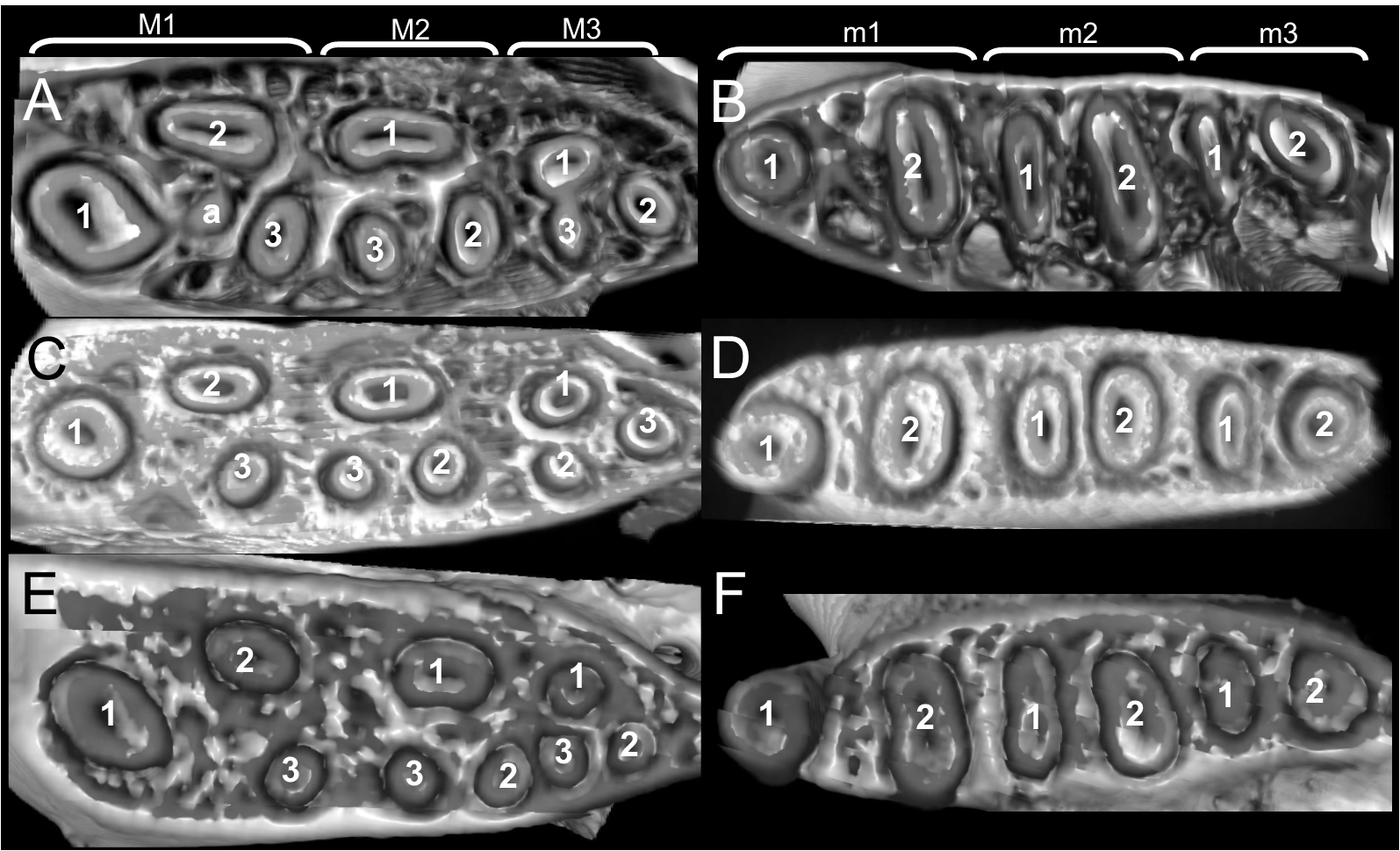

Supplement: Supplemental Information 7 — A, B, Pattonimus ecominga sp. nov. (MECN 5928, holotype); C, D, Mindomys hammondi (BMNH 13.10.24.58, holotype); and E, F, Nephelomys auriventer (MECN 5812). Abbreviations: a = accessory root; 1, 2, 3 = main roots; M1, M2, M3 = upper molars; m1, m2, m3 = lower molars. [file peerj-08-10247-s007.png]

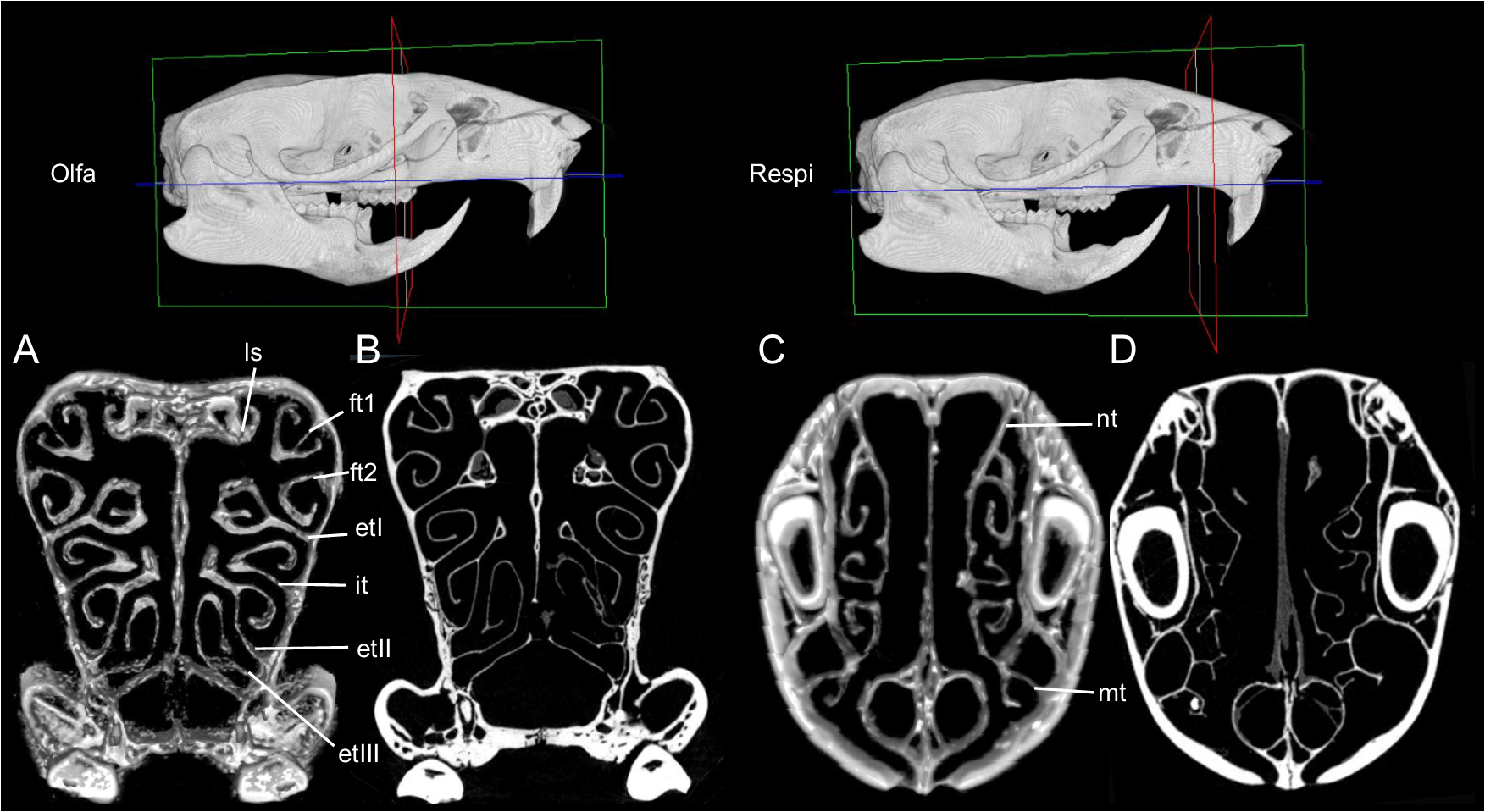

Supplement: Supplemental Information 10 — A, C, Pattonimus ecominga sp. nov. (MECN 5928, holotype), and B, D, Mindomys hammondi (BMNH 13.10.24.58, holotype). Abbreviations: etI = ethmoturbinal I, etII = ethmoturbinal II, etIII = ethmoturbinal III, ft1 = frontoturbinal 1, ft2 = frontoturbinal 2, it = interturbinal, ls = lamina semicircularis, mt = maxilloturbinal, nt = nasoturbinal, Olfa = olfactory turbinals, Respi = respiratory turbinals. [file peerj-08-10247-s010.png]

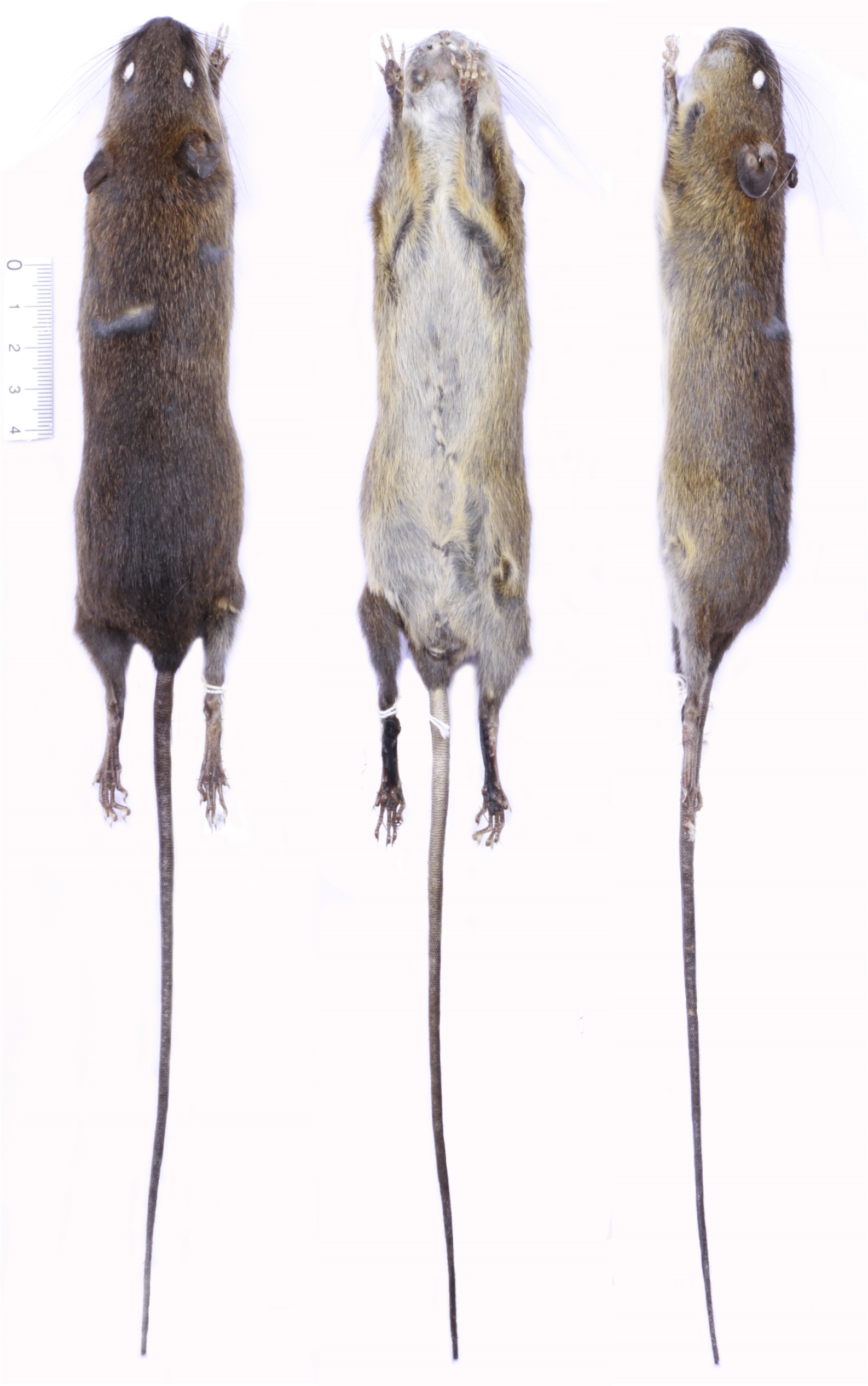

Supplement: Supplemental Information 11 [file peerj-08-10247-s011.jpg]

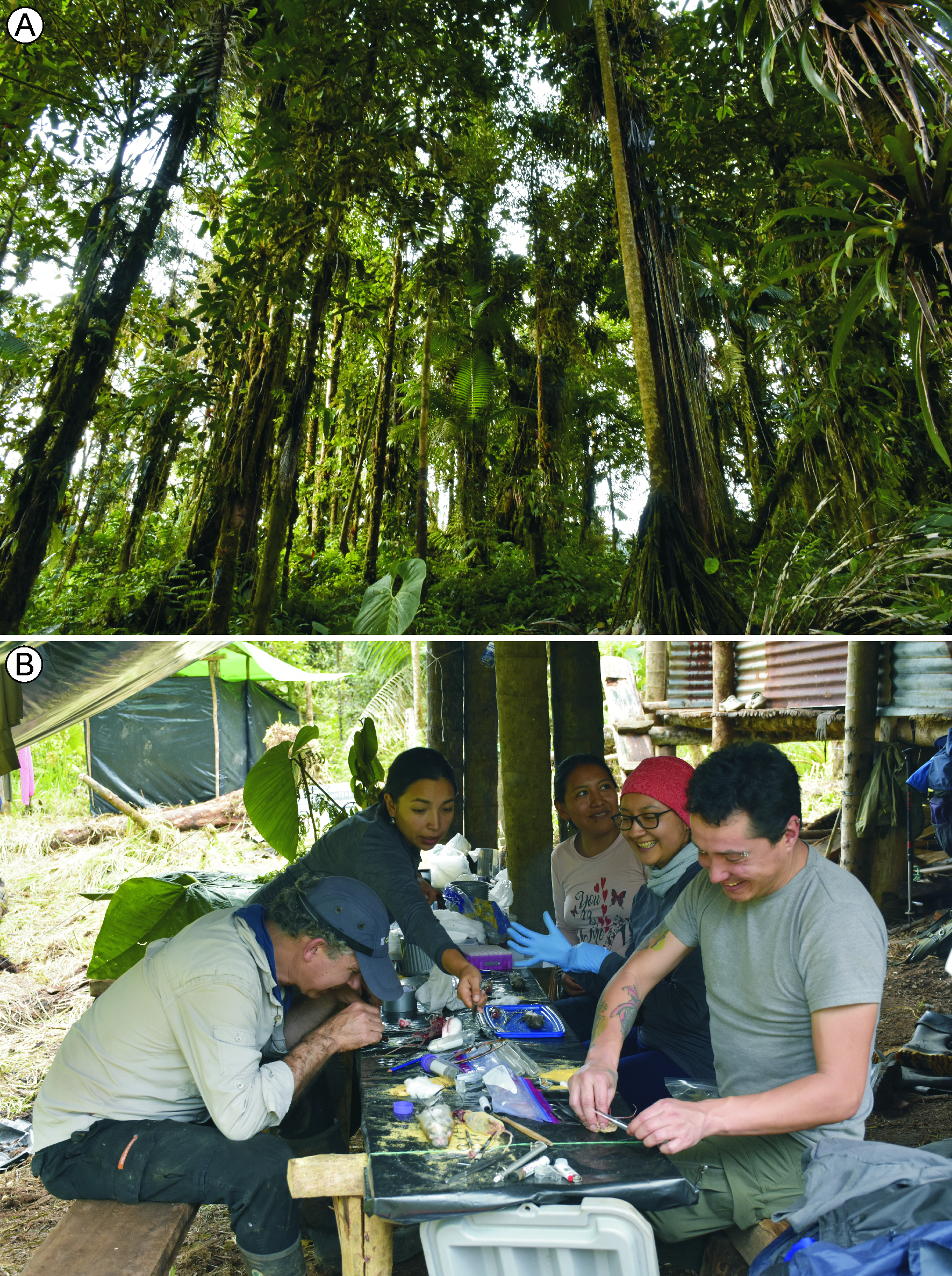

Supplement: Supplemental Information 12 — A, Cloud forest at Reserva Drácula, habitat of Pattonimus ecominga sp. nov., and B, the team (from left to right, U. Pardiñas, R. García, J. Curay, S. Pozo, and C. Nivelo) processing the harvest at Drácula basecamp (photos: J. Brito). [file peerj-08-10247-s012.jpg]

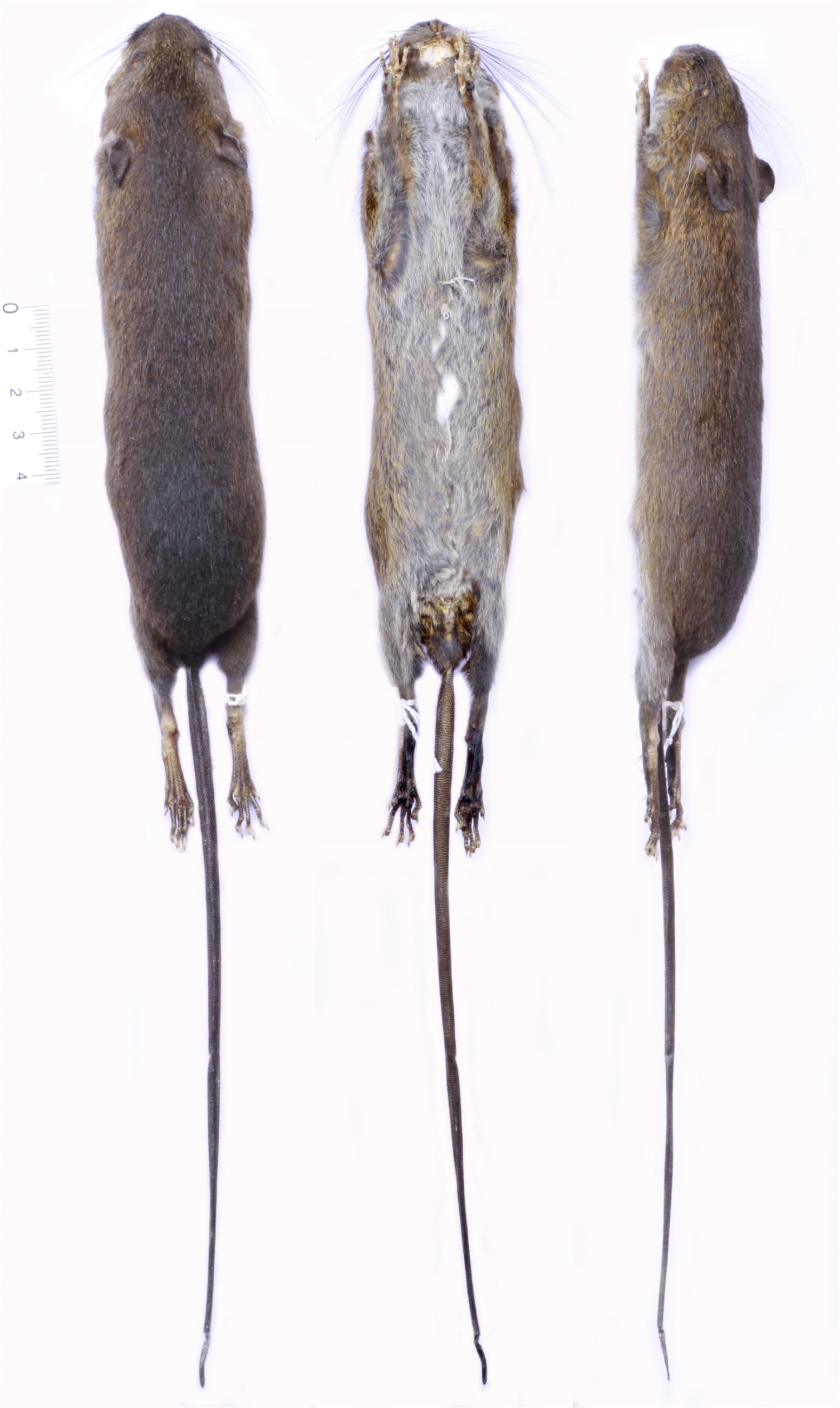

Supplement: Supplemental Information 14 — Pattonimus musseri sp. nov. (Reserva Río Manduriacu, Imbabura, Ecuador): dry skin in dorsal, ventral, and lateral views (MEPN 12605, holotype). [file peerj-08-10247-s014.jpg]

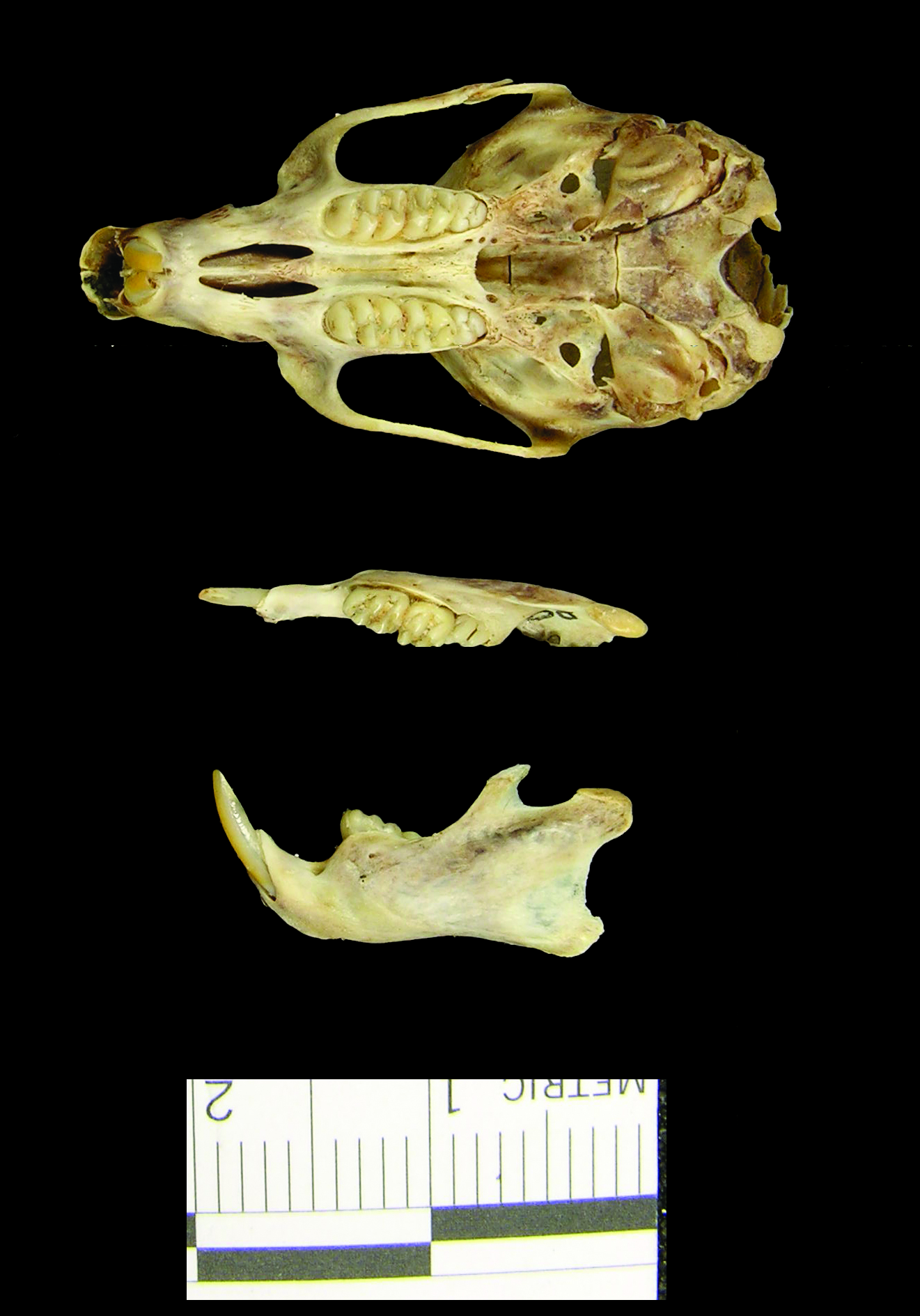

Supplement: Supplemental Information 15 — Pattonimus sp. from Reserva Otonga (QCAZ 8720; Cotopaxi, Ecuador): cranium in ventral view (top), and hemimandible in dorsal and labial views (bottom). [file peerj-08-10247-s015.jpg]
