# Supplementary material for: A new genus of oryzomyine rodents (Cricetidae, Sigmodontinae) with three new species from montane cloud forests, western Andean cordillera of Colombia and Ecuador
Source: PeerJ. 2020 Nov 10;8:e10247. doi: 10.7717/peerj.10247 (PMC7664470; doi:10.7717/peerj.10247)
Supplement: Supplemental Information 2 [file peerj-08-10247-s002.docx]

**Supplemental Information S2:** List of studied specimens.

Studied specimens belong to the following mammal collections: BM, The Natural History Museum, London, United Kingdom; MECN, Instituto Nacional de Biodiversidad, Quito, Ecuador; MEPN, Museo de la Escuela Politécnica Nacional, Quito, Ecuador; QCAZ, Museo Pontificia Universidad Católica del Ecuador, Quito, Ecuador; ROM, Royal Ontario Museum, Toronto, Canada; UMMZ, Museum of Zoology, University of Michigan, Ann Arbor, Michigan, USA; and ZFMK, Zoologisches Forschungsmuseum Alexander Koenig, Bonn, Germany. Specimens marked with an * are holotypes.

***Mindomys hammondi*** (n = 8): Pichincha, San Miguel de los Bancos, Mindo (BM 13.10.24.58*, 34.9.10.212, 34.9.10.210, 13.10.24.57, 34.9.10.213, 34.9.10.211, 34.9.10.209, UMMZ 155827); Esmeraldas, Alto Tambo (ROM 105820).

***Nephelomys albigularis*** (n = 24): Ecuador, Bolívar, Quebrada de Pistud (MECN 527, 528; Cruz de Liso (MECN 578, 579, 580, 581, 583); El Oro, Zaruma, Chivaturco (MECN 4782, 4784, 4785, 4786, 4787, 4788, 4798, 4799, 4803, 4804), Chilla, Chilla Cocha (MECN 4813, 4817, 4823, 4824, 4825, 4833, 4834).

***Nephelomys auriventer*** (n = 5): Ecuador, Morona Santiago, Morona, Sardinayacu (MECN 3797), Mendez, Kutukú (MECN 5812, 5813, 5816), Logroño, Yapit (MEPN 12214).

***Nephelomys nimbosus*** (n = 9): Ecuador, Morona Santiago, Guabisai (MECN 4322-4326), Tinguichaca (MECN 3803); Sambalán (MECN 4325); Tungurahua, Baños, Cerro Candelaria (MECN 5010), Río Cristal (MECN 6090).

***Nephelomys moerex*** (n = 26): Ecuador, Cotopaxi, Otonga (MECN 1084, 1086); Pichincha, Reserva Pahuma (MECN 2480, 2485, 2513, 2515, 2516, 2517), Reserva Verdecocha (MECN 2538, 2539, 2541, 2592), Reserva Maquipucuna (MECN 2545, 2605), Reserva Vellavista (MECN 2816, 2817, 2818, 2819, 2820, 2824, 2826, 2827, 2829, 2830, 2832, 2835), Mindo (ZFMK 1959-0211-sk).

***Pattonimus ecominga*** (n = 22): Ecuador, Carchi, Reserva Drácula, Gualpilal (MECN 5927, 5928*, 6034), Pailón Alto (MECN 6017) Km. 18, Gualpi (MECN 5293, 5297, 5298, 5304, 5308, 5309, 5310, 5325, 5326, 5382, 6019, 6020, 6025, 6040, 6041, 6042, 6043), Km. 14 (MECN 4991).

***Pattonimus musseri*** (n = 4): Ecuador, Imbabura, Reserva Río Manduriacu (MEPN 12586, 12587, 12593, 12605*).

***Pattonimus*** sp. (n = 3): Ecuador, Cotopaxi, Otonga (QCAZ 8720). Colombia, Nariño, Reserva del río Ñambi (ICN 13663, 21487).

***Sigmodontomys alfari*** (n = 2): Ecuador, Carchi, Reserva Drácula (MECN 6021, 6022).

***Tanyuromys thomasleei*** (n = 4): Ecuador, Carchi, Reserva Drácula (MECN 4740, 5938); Imbabura, Reserva Río Manduriacu (MEPN 12606); Pichincha, La Titania (MECN 3407).
