# Supplementary material for: A new genus of oryzomyine rodents (Cricetidae, Sigmodontinae) with three new species from montane cloud forests, western Andean cordillera of Colombia and Ecuador
Source: PeerJ. 2020 Nov 10;8:e10247. doi: 10.7717/peerj.10247 (PMC7664470; doi:10.7717/peerj.10247)
Supplement: Supplemental Information 8 — Individuals are labelled by museum collection numbers with the addition of “e” = Reserva Drácula, and “m” = Reserva Río Manduriacu (terminals with * denote holotypes). [file peerj-08-10247-s008.docx]

Supplemental Information S8. Results of UPGMA clustering of Mahalanobis distances among two geographic samples (molar measurements transformed to natural logarithms) of the new genus; individuals are labelled by museum collection numbers with the addition of “e” = Reserva Drácula, and “m” = Reserva Río Manduriacu (terminals with * denote holotypes).
