# Supplementary material for: A new genus of oryzomyine rodents (Cricetidae, Sigmodontinae) with three new species from montane cloud forests, western Andean cordillera of Colombia and Ecuador
Source: PeerJ. 2020 Nov 10;8:e10247. doi: 10.7717/peerj.10247 (PMC7664470; doi:10.7717/peerj.10247)
Supplement: Supplemental Information 9 — Original matrix composed of 38 external and craniodental measurements, transformed to their natural logarithms. [file peerj-08-10247-s009.docx]

Supplemental Information S9. PCA including 10 individuals from Reserva Drácula (labelled with “e”) and 2 individuals from Reserva Río Manduriacu (labelled with “m”); original matrix composed of 38 external and craniodental measurements. transformed to their natural logarithms.

|  | Factor 1 | Factor 2 | Factor 3 |
| --- | --- | --- | --- |
| Head and body length | -0.064467 | -0.049108 | 0.025339 |
| Tail length | -0.076911 | -0.017447 | -0.013918 |
| Hind foot length | -0.026457 | -0.005365 | 0.020426 |
| Ear length | -0.022498 | -0.078155 | 0.025011 |
| Length of longest mystacial vibrissae | -0.013456 | 0.019565 | -0.037730 |
| Length of longest genal vibrissae | -0.021236 | 0.003077 | -0.047828 |
| Occipitonasal length | -0.042982 | 0.000838 | -0.011349 |
| Condylo-incisive length | -0.048009 | 0.001004 | -0.012746 |
| Length of upper diastema | -0.058632 | 0.005650 | -0.008209 |
| Crown length of maxillary toothrow | -0.000984 | -0.000527 | 0.001657 |
| Length of incisive foramen | -0.079093 | 0.030333 | 0.017660 |
| Breadth of incisive foramina | -0.043736 | 0.046535 | 0.014791 |
| Breadth of M1 | 0.003210 | -0.007057 | -0.015380 |
| Breadth of rostrum | -0.050036 | -0.006476 | -0.009196 |
| Length of nasals | -0.053875 | -0.008056 | -0.021725 |
| Length of palatal bridge | -0.031648 | -0.026002 | -0.036144 |
| Breadth of bony palate | -0.030715 | 0.039053 | 0.028259 |
| Least interorbital breadth | -0.005571 | 0.005483 | -0.001570 |
| Zygomatic breadth | -0.041357 | 0.000618 | -0.008244 |
| Breadth of zygomatic plate | -0.040084 | -0.011348 | -0.016654 |
| Lambdoidal breadth | -0.017382 | -0.003584 | 0.005005 |
| Orbital fossa length | -0.039838 | 0.002658 | -0.007233 |
| Bular breadth | -0.014798 | 0.003066 | -0.000652 |
| Length of mandible | -0.050299 | -0.000205 | -0.001290 |
| Crown length of mandibular toothrow | 0.001832 | 0.011324 | -0.006995 |
| Length of lower diastema | -0.021827 | -0.004568 | 0.017539 |
| Length M1 | -0.010144 | 0.022788 | -0.003389 |
| Width M1 | -0.008832 | 0.003982 | -0.004678 |
| Length M2 | -0.040662 | 0.042336 | 0.044798 |
| Width M2 | -0.001939 | -0.013730 | 0.006130 |
| Length M3 | 0.006389 | -0.006293 | 0.002851 |
| Width M3 | -0.004855 | -0.015755 | 0.008563 |
| Length m1 | -0.006728 | 0.028843 | -0.035414 |
| Width m1 | -0.002095 | 0.006831 | -0.032914 |
| Length M2 | -0.017290 | 0.012005 | 0.010610 |
| Width m2 | -0.006417 | -0.014588 | -0.016338 |
| Length M3 | 0.014768 | 0.007800 | -0.042259 |
| Width m3 | -0.006125 | -0.016750 | -0.004763 |

PCA including 10 individuals from Reserva Drácula (labelled with “e”) and 2 individuals from Reserva Río Manduriacu (labelled with “m”); original matrix composed of the 5 craniodental measurements showing significant differences. transformed to their natural logarithms.

| Analysis of Variance (Spreadsheet1) Marked effects are significant at p < .05000 | | | | | |  |  |  |
| --- | --- | --- | --- | --- | --- | --- | --- | --- |
| e vs m | SS - Effect | df - Effect | MS - Effect | SS - Error | df - Error | MS - Error | F | p |
| Head and body length | 0.001713 | 1 | 0.001713 | 0.085772 | 10 | 0.008577 | 0.19973 | 0.664463 |
| Tail length | 0.008172 | 1 | 0.008172 | 0.072968 | 10 | 0.007297 | 1.11999 | 0.314815 |
| Hind foot length | 0.000132 | 1 | 0.000132 | 0.024464 | 10 | 0.002446 | 0.05388 | 0.821121 |
| Ear length | 0.017008 | 1 | 0.017008 | 0.072946 | 10 | 0.007295 | 2.33158 | 0.157761 |
| Length of longest mystacial vibrissae | 0.006257 | 1 | 0.006257 | 0.027118 | 10 | 0.002712 | 2.30746 | 0.159718 |
| Length of longest genal vibrissae | 0.010474 | 1 | 0.010474 | 0.07942 | 10 | 0.007942 | 1.31877 | 0.277541 |
| Occipitonasal length | 0.007748 | 1 | 0.007748 | 0.017189 | 10 | 0.001719 | 4.50771 | 0.059707 |
| Condylo-incisive length | 0.009546 | 1 | 0.009546 | 0.019001 | 10 | 0.0019 | 5.02386 | 0.048888 |
| Length of upper diastema | 0.012314 | 1 | 0.012314 | 0.030724 | 10 | 0.003072 | 4.00796 | 0.073145 |
| Crown length of maxillary toothrow | 0.000014 | 1 | 0.000014 | 0.002729 | 10 | 0.000273 | 0.05194 | 0.82431 |
| Length of incisive foramen | 0.015091 | 1 | 0.015091 | 0.092654 | 10 | 0.009265 | 1.6288 | 0.230712 |
| Breadth of incisive foramina | 0.017884 | 1 | 0.017884 | 0.050083 | 10 | 0.005008 | 3.57093 | 0.088107 |
| Breadth of M1 | 0.000001 | 1 | 0.000001 | 0.005672 | 10 | 0.000567 | 0.00178 | 0.967183 |
| Breadth of rostrum | 0.009425 | 1 | 0.009425 | 0.025339 | 10 | 0.002534 | 3.71956 | 0.082623 |
| Length of nasals | 0.007433 | 1 | 0.007433 | 0.035223 | 10 | 0.003522 | 2.11019 | 0.176967 |
| Length of palatal bridge | 0.00134 | 1 | 0.00134 | 0.036084 | 10 | 0.003608 | 0.37136 | 0.555853 |
| Breadth of bony palate | 0.026688 | 1 | 0.026688 | 0.043957 | 10 | 0.004396 | 6.07133 | 0.033446 |
| Least interorbital breadth | 0.001589 | 1 | 0.001589 | 0.003993 | 10 | 0.000399 | 3.97902 | 0.074033 |
| Zygomatic breadth | 0.005284 | 1 | 0.005284 | 0.015534 | 10 | 0.001553 | 3.40172 | 0.094916 |
| Breadth of zygomatic plate | 0.001638 | 1 | 0.001638 | 0.038771 | 10 | 0.003877 | 0.42239 | 0.53039 |
| Lambdoidal breadth | 0.000596 | 1 | 0.000596 | 0.00558 | 10 | 0.000558 | 1.06876 | 0.325579 |
| Orbital fossa length | 0.004801 | 1 | 0.004801 | 0.020584 | 10 | 0.002058 | 2.3324 | 0.157696 |
| Bular breadth | 0.00169 | 1 | 0.00169 | 0.003714 | 10 | 0.000371 | 4.54942 | 0.05873 |
| Length of mandible | 0.009557 | 1 | 0.009557 | 0.020028 | 10 | 0.002003 | 4.77197 | 0.053836 |
| Crown length of mandibular toothrow | 0.00129 | 1 | 0.00129 | 0.002025 | 10 | 0.000203 | 6.37207 | 0.030164 |
| Length of lower diastema | 0.000036 | 1 | 0.000036 | 0.027573 | 10 | 0.002757 | 0.01291 | 0.911772 |
| Length M1 | 0.006726 | 1 | 0.006726 | 0.023123 | 10 | 0.002312 | 2.90885 | 0.118907 |
| Width M1 | 0.001879 | 1 | 0.001879 | 0.003852 | 10 | 0.000385 | 4.87759 | 0.051689 |
| Length M2 | 0.026065 | 1 | 0.026065 | 0.046461 | 10 | 0.004646 | 5.61004 | 0.039374 |
| Width M2 | 0.000454 | 1 | 0.000454 | 0.009884 | 10 | 0.000988 | 0.45984 | 0.513085 |
| Length M3 | 0.000276 | 1 | 0.000276 | 0.047313 | 10 | 0.004731 | 0.05831 | 0.814074 |
| Width M3 | 0.001825 | 1 | 0.001825 | 0.004106 | 10 | 0.000411 | 4.44437 | 0.061231 |
| Length m1 | 0.020216 | 1 | 0.020216 | 0.020081 | 10 | 0.002008 | 10.06699 | 0.009939 |
| Width m1 | 0.00388 | 1 | 0.00388 | 0.017542 | 10 | 0.001754 | 2.2118 | 0.167795 |
| Length M2 | 0.002373 | 1 | 0.002373 | 0.010782 | 10 | 0.001078 | 2.20053 | 0.168781 |
| Width m2 | 0.000211 | 1 | 0.000211 | 0.012501 | 10 | 0.00125 | 0.16903 | 0.689649 |
| Length M3 | 0.000821 | 1 | 0.000821 | 0.031068 | 10 | 0.003107 | 0.26437 | 0.618302 |
| Width m3 | 0.000001 | 1 | 0.000001 | 0.011978 | 10 | 0.001198 | 0.00121 | 0.972959 |

| PC | Eigenvalue | % variance |
| --- | --- | --- |
| 1 | 0.00732611 | 52.622 |
| 2 | 0.00346816 | 24.911 |
| 3 | 0.00217041 | 15.59 |
| 4 | 0.00085603 | 6.1487 |
| 5 | 0.00010132 | 0.72778 |

PCA including 10 individuals from Reserva Drácula (labelled with “e”). 2 individuals from Reserva Río Manduriacu (labelled with “m”). and 2 individuals from southern Colombia (labelled with “c”); original matrix composed of the 7 craniodental measurements showing significant differences. transformed to their natural logarithms.

| Analysis of Variance (Spreadsheet6) Marked effects are significant at p < .05000 | | | | | |  |  |  |
| --- | --- | --- | --- | --- | --- | --- | --- | --- |
| Groups c. m y e | SS | df | MS | SS | df | MS | F | p |
| Head and body length | 0.022675 | 2 | 0.011337 | 0.086193 | 11 | 0.007836 | 1.446886 | 0.276789 |
| Tail length | 0.022145 | 2 | 0.011072 | 0.073210 | 11 | 0.006655 | 1.663669 | 0.233749 |
| Hind foot length | 0.005192 | 2 | 0.002596 | 0.024716 | 11 | 0.002247 | 1.155323 | 0.350400 |
| Condylo-incisive length | 0.015044 | 2 | 0.007522 | 0.019046 | 11 | 0.001731 | 4.344254 | 0.040690 |
| Length of upper diastema | 0.035446 | 2 | 0.017723 | 0.032166 | 11 | 0.002924 | 6.060833 | 0.016809 |
| Crown length of maxillary toothrow | 0.000045 | 2 | 0.000023 | 0.002892 | 11 | 0.000263 | 0.086448 | 0.917800 |
| Breadth of incisive foramina | 0.079070 | 2 | 0.039535 | 0.059603 | 11 | 0.005418 | 7.296289 | 0.009617 |
| Breadth of M1 | 0.000012 | 2 | 0.000006 | 0.005818 | 11 | 0.000529 | 0.011711 | 0.988370 |
| Breadth of rostrum | 0.019073 | 2 | 0.009537 | 0.026187 | 11 | 0.002381 | 4.005881 | 0.049322 |
| Length of nasals | 0.031738 | 2 | 0.015869 | 0.036027 | 11 | 0.003275 | 4.845196 | 0.030969 |
| Length of palatal bridge | 0.002439 | 2 | 0.001220 | 0.037202 | 11 | 0.003382 | 0.360642 | 0.705174 |
| Least interorbital breadth | 0.002819 | 2 | 0.001410 | 0.005545 | 11 | 0.000504 | 2.796086 | 0.104278 |
| Zygomatic breadth | 0.013000 | 2 | 0.006500 | 0.015680 | 11 | 0.001425 | 4.559765 | 0.036121 |
| Breadth of zygomatic plate | 0.029824 | 2 | 0.014912 | 0.038821 | 11 | 0.003529 | 4.225295 | 0.043504 |
| Orbital fossa length | 0.012667 | 2 | 0.006334 | 0.020625 | 11 | 0.001875 | 3.377881 | 0.071828 |
